# Lower Admission CALLY Index Is Associated with Contrast-Associated Acute Kidney Injury in Patients with STEMI Undergoing Primary PCI

**DOI:** 10.3390/medicina62091811

**Published:** 2026-09-20

**Authors:** Emre Eynel, Direnç Yılmaz, Arslan Nyyazov, Eren Eynel, Ibrahim Kocayiğit, Muhammed Necati Murat Aksoy

**Affiliations:** 1 Department of Cardiology, Sakarya University Training and Research Hospital, 54100 Sakarya, Turkey; emreeynel@gmail.com (E.E.); direnc.yilmaz@saglik.gov.tr (D.Y.); 2 Department of Cardiology, Sakarya University School of Medicine, 54100 Sakarya, Turkey; niyazzov_93@list.ru (A.N.); mnaksoy@sakarya.edu.tr (M.N.M.A.); 3 Department of Nephrology, Diyarbakır Gazi Yaşargil Training and Research Hospital, 21070 Diyarbakır, Turkey; ereneynel@hotmail.com

**Keywords:** CALLY index, contrast-associated acute kidney injury, ST-segment elevation myocardial infarction, primary percutaneous coronary intervention, inflammatory biomarkers

## Abstract

*Background and Objectives*: The C-reactive protein–albumin–lymphocyte (CALLY) index integrates inflammatory, nutritional, and immune information, but its relationship with contrast-associated acute kidney injury (CA-AKI) in patients with ST-segment elevation myocardial infarction (STEMI) treated with primary percutaneous coronary intervention (PCI) remains uncertain. This study evaluated the association between admission CALLY and CA-AKI and compared its discriminatory performance with other inflammatory indices. *Materials and Methods*: This retrospective, single-center cohort included 903 patients with STEMI who underwent primary PCI between January 2018 and December 2024. CA-AKI was defined as an increase in serum creatinine of ≥0.3 mg/dL or ≥1.5-fold from baseline within 48–72 h after contrast exposure. Multivariable logistic regression assessed the independent association between CALLY and CA-AKI. Receiver operating characteristic analyses compared CALLY with the C-reactive protein-to-albumin ratio (CAR), C-reactive protein (CRP), neutrophil-to-lymphocyte ratio (NLR), and systemic immune-inflammation index (SII). *Results*: CA-AKI occurred in 139 patients (15.4%). Median CALLY was lower in patients with than without CA-AKI (0.635 [IQR, 0.193–1.529] vs. 1.536 [IQR, 0.891–2.356]; *p* < 0.001). Each 50% decrease in CALLY was independently associated with higher odds of CA-AKI (adjusted OR, 1.587; 95% CI, 1.367–1.843; *p* < 0.001). CALLY yielded an AUC of 0.734 (95% CI, 0.683–0.782). Its discrimination was greater than that of CRP, NLR, and SII but was not significantly different from that of CAR (AUC, 0.707; DeLong *p* = 0.163). *Conclusions*: Lower admission CALLY was independently associated with CA-AKI and showed moderate discriminatory ability. Prospective multicenter studies are needed to validate these findings and establish clinical utility.

## 1. Introduction

Contrast-associated acute kidney injury (CA-AKI) is an important clinical complication following coronary angiography and percutaneous coronary intervention (PCI) [1]. It is particularly relevant in patients with ST-segment elevation myocardial infarction (STEMI) undergoing primary PCI, in whom several risk factors often coexist, including advanced age, diabetes mellitus, impaired baseline renal function, hemodynamic instability, extensive coronary artery disease, and substantial contrast exposure. In a recent meta-analysis of patients with STEMI undergoing primary PCI, CA-AKI occurred in approximately 12% of patients and was associated with various established clinical and procedural risk factors [2]. In addition, the severity of acute kidney injury after contrast-based cardiovascular procedures has been linked to adverse outcomes, including the requirement for renal replacement therapy, rehospitalization, and mortality [3].

Although pre-existing renal dysfunction and procedure-related factors are well-established determinants of CA-AKI, growing evidence suggests that systemic inflammation, immune status, and nutritional status may also contribute to its development. In patients with STEMI undergoing primary PCI, an elevated neutrophil-to-lymphocyte ratio (NLR) has been independently associated with CA-AKI [4]. Similarly, the C-reactive protein-to-albumin ratio (CAR), which combines an inflammatory marker with a parameter affected by both systemic inflammation and nutritional status, has been associated with acute kidney injury after primary PCI [5]. The prognostic nutritional index, which incorporates serum albumin and lymphocyte count, has also shown an inverse and independent association with CA-AKI in patients with STEMI [6]. Other composite inflammatory indices, including the systemic immune-inflammation index (SII) and lymphocyte-to-C-reactive protein ratio, have likewise been associated with post-contrast renal injury in STEMI populations [7,8].

The C-reactive protein–albumin–lymphocyte (CALLY) index is a composite biomarker that integrates serum C-reactive protein, albumin, and lymphocyte count into a single measure reflecting systemic inflammation, nutritional status, and immune response. It was initially introduced by Iida et al. as a prognostic marker in patients undergoing hepatectomy for hepatocellular carcinoma [9]. More recently, its prognostic value has been investigated in cardiovascular disease. In patients with STEMI undergoing primary PCI, a lower CALLY index has been associated with in-hospital mortality, supporting its potential relevance in acute myocardial infarction [10]. Regarding renal outcomes, the CALLY index has recently been evaluated in patients with stable angina and NSTEMI undergoing coronary procedures, although the strength of the association varied across studies [11,12].

Nevertheless, the association between the CALLY index and CA-AKI has not been sufficiently characterized in patients with STEMI undergoing primary PCI. Given that CALLY integrates inflammatory, nutritional, and immune information, its evaluation in this setting may be clinically relevant. The present study aimed to evaluate the association between the admission CALLY index and CA-AKI in this population and to compare its discriminatory performance with that of CAR, CRP, NLR, and SII.

## 2. Materials and Methods

### 2.1. Study Design and Setting

This single-center, retrospective, observational cohort study screened patients admitted with ST-segment elevation myocardial infarction (STEMI) at Sakarya University Training and Research Hospital between January 2018 and December 2024.

The study protocol was approved by the Ethics Committee of Sakarya University (approval date: 16 March 2026; decision no. E-43012747-050.04-572572-205). The requirement for written informed consent was waived because of the retrospective nature of the study. The study was conducted in accordance with the principles of the Declaration of Helsinki. The study was reported in accordance with the Strengthening the Reporting of Observational Studies in Epidemiology (STROBE) statement.

### 2.2. Eligibility Criteria

Patients aged 18–90 years who underwent emergency coronary angiography with the intention of primary PCI for the index STEMI were assessed for eligibility.

Eligibility required angiographically documented obstructive coronary artery disease involving at least one major epicardial coronary artery and the availability of admission laboratory measurements to calculate the C-reactive protein–albumin–lymphocyte (CALLY) index, including C-reactive protein (CRP), serum albumin, and lymphocyte count. A serum creatinine measurement obtained before contrast administration and at least one follow-up serum creatinine measurement within 48–72 h after contrast exposure were also required for ascertainment of the primary endpoint. Patients were included only when the clinical, laboratory, angiographic, and procedural data necessary for the study analyses were available.

Patients were excluded if they had systemic inflammatory or autoimmune disease, pre-existing decompensated heart failure unrelated to the index STEMI, liver disease or active hepatitis, malignancy, chronic kidney disease with an estimated glomerular filtration rate (eGFR) < 30 mL/min/1.73 m^2^, sepsis, pregnancy, or active major bleeding at presentation. Other exclusion criteria were chronic corticosteroid or systemic anti-inflammatory therapy; ongoing pre-admission use of known nephrotoxic drugs, including nonsteroidal anti-inflammatory drugs, aminoglycosides, or amphotericin B; and additional iodinated contrast exposure during the 48–72-h CA-AKI ascertainment window, including repeat PCI or contrast-enhanced imaging. Patients presenting more than 12 h after symptom onset, those treated with fibrinolytic therapy, and those with sudden cardiac arrest at presentation were also excluded. Death during the index procedure or within the first 48 h of hospitalization was an exclusion criterion because the post-contrast serum creatinine measurements required for CA-AKI assessment could not be completed. Patients who underwent emergency or elective coronary artery bypass grafting during the index hospitalization were also excluded.

### 2.3. Clinical Definitions

STEMI was diagnosed according to established diagnostic criteria. Eligible patients presented with ischemic chest pain lasting >30 min within 12 h of symptom onset and electrocardiographic evidence of ST-segment elevation measured at the J point in at least two contiguous leads. ST-segment elevation was defined as ≥2.5 mm in men aged <40 years, ≥2.0 mm in men aged ≥40 years, and ≥1.5 mm in women in leads V2–V3, or ≥1.0 mm in other contiguous leads. Patients with a new bundle branch block and a clinical presentation suggestive of acute coronary occlusion were also evaluated in the context of STEMI. Posterior myocardial infarction was considered in the presence of ST-segment depression in leads V1–V3 with compatible electrocardiographic findings, with additional assessment of posterior leads V7–V9 for ST-segment elevation ≥ 0.5 mm when clinically indicated [13,14].

Hypertension, diabetes mellitus, previous cerebrovascular disease, and known coronary artery disease were identified from medical history, previous hospital records, and/or ongoing medical treatment. Hypertension was defined as previously documented systolic blood pressure ≥ 140 mmHg and/or diastolic blood pressure ≥ 90 mmHg on at least two separate measurements or current use of antihypertensive medication. Diabetes mellitus was defined as fasting plasma glucose ≥ 126 mg/dL, random plasma glucose ≥ 200 mg/dL, glycated hemoglobin ≥ 6.5%, or current antidiabetic medication use. Dyslipidemia was defined by a documented history of lipid-lowering medication use before the index hospital admission. Smoking was defined as regular tobacco use within the preceding six months. Pre-admission angiotensin-converting enzyme inhibitor or angiotensin II receptor blocker (ACEI/ARB) use was defined as documented regular use of either medication class before the index hospital admission.

### 2.4. Data Collection

Demographic, clinical, laboratory, echocardiographic, angiographic, procedural, and in-hospital outcome data were retrospectively obtained from electronic medical records, patient files, catheterization laboratory records, and the hospital information system.

Recorded demographic and clinical variables included age, sex, hypertension, diabetes mellitus, smoking status, dyslipidemia, previous cerebrovascular disease, known coronary artery disease, Killip class at presentation, pre-admission ACEI/ARB use, and the P2Y12 inhibitor administered during the index hospitalization.

### 2.5. Laboratory Measurements and Calculation of Inflammatory and Nutritional Indices

Laboratory measurements obtained as part of routine clinical care at hospital admission and during subsequent follow-up were retrospectively reviewed. Hematologic parameters were analyzed using an automated hematology analyzer (Beckman Coulter LH 780; Mervue, Galway, Ireland). Admission laboratory parameters included serum creatinine, albumin, CRP, hemoglobin, white blood cell count, neutrophil count, lymphocyte count, and platelet count. Serum creatinine and albumin were measured using spectrophotometric methods, whereas standard CRP was measured using an immunoturbidimetric method on an Olympus AU5800 autoanalyzer (Beckman Coulter, Brea, CA, USA).

The serum creatinine value obtained before coronary angiography and contrast administration was considered the baseline serum creatinine level. Serum creatinine measurements obtained within 48–72 h after contrast exposure were reviewed, and the highest value recorded during this period was defined as the peak post-contrast serum creatinine level.

Baseline eGFR was calculated using the 2021 Chronic Kidney Disease Epidemiology Collaboration (CKD-EPI) creatinine equation [15].

The lower reporting threshold for standard CRP at the study-center laboratory was 3 mg/L. CRP concentrations reported under this threshold were assigned a value of 3 mg/L for statistical analyses and for calculation of CRP-based indices. This substitution was applied to 4 of 903 patients (0.4%).

The CALLY index was calculated using the formula originally described by Iida et al. [9]:

CALLY index = [Albumin (g/dL) × absolute lymphocyte count (/µL)]/[CRP (mg/dL) × 10^4^]

Because serum albumin, lymphocyte count, and CRP were routinely reported at our institution in g/L, ×10^9^/L, and mg/L, respectively, laboratory values were converted to the units applied in the original CALLY formula before calculation.

The C-reactive protein-to-albumin ratio (CAR) was calculated from admission CRP and serum albumin measurements as follows [5]:

CAR = CRP (mg/L)/Albumin (g/L)

The neutrophil-to-lymphocyte ratio (NLR) was calculated as follows:

NLR = Neutrophil count/Lymphocyte count

The systemic immune-inflammation index (SII) was calculated according to the previously described formula [16]:

SII = Platelet count × Neutrophil count/Lymphocyte count

All inflammatory and nutritional indices were calculated using laboratory measurements obtained at hospital admission and before primary PCI.

### 2.6. Echocardiographic Assessment

All patients underwent transthoracic echocardiographic examination using a commercially available ultrasound system (Vivid E9; GE Healthcare, Chicago, IL, USA) within the first 24 h of hospital admission. Left ventricular ejection fraction (LVEF) was assessed using the biplane Simpson method from apical four- and two-chamber views when technically feasible. The earliest echocardiographic examination performed during the index hospitalization was used for the study analysis.

### 2.7. Primary PCI and Angiographic Assessment

Emergency coronary angiography and primary PCI were performed in accordance with standard clinical practice and at the discretion of the treating interventional cardiologist.

Before the procedure, patients received a loading dose of acetylsalicylic acid (300 mg) together with a P2Y12 inhibitor, consisting of clopidogrel (600 mg), prasugrel (60 mg), or ticagrelor (180 mg), as clinically appropriate.

A nonionic, low-osmolar iodinated contrast medium, iohexol (Omnipaque^®^, GE HealthCare AS, Oslo, Norway), was used during coronary angiography and primary PCI. The total volume of contrast medium administered during the index procedure was recorded for each patient.

Routine prophylactic preprocedural intravenous hydration for CA-AKI prevention was not administered because the emergency nature of primary PCI did not allow for a standardized preprocedural hydration protocol.

The infarct-related artery (IRA) was identified based on angiographic findings and classified as the left anterior descending artery (LAD), left circumflex artery (LCx), right coronary artery (RCA), or other. Cases in which a side branch of a major epicardial coronary artery was identified as the infarct-related artery were grouped into the “other” category.

Obstructive coronary artery disease was defined as a visually estimated luminal diameter stenosis of ≥50% in a major epicardial coronary artery [17]. The number of diseased major epicardial coronary vessels was determined according to the presence of obstructive coronary artery disease in the LAD, LCx, and RCA. It was classified as one-, two-, or three-vessel disease. Side-branch lesions were not counted as additional diseased vessels.

The SYNTAX score was calculated from the diagnostic coronary angiogram using the standard SYNTAX scoring methodology to quantify the anatomical extent and complexity of coronary artery disease [17]. Angiographic lesions with ≥50% diameter stenosis in eligible coronary segments were considered for scoring according to the standard methodology. The SYNTAX score and angiographic no-reflow assessments were performed retrospectively and independently by two cardiologists experienced in interventional cardiology who were blinded to the patients’ CALLY values and CA-AKI status. Disagreements were resolved by consensus.

Primary PCI was performed with stent implantation or, in selected patients, with balloon angioplasty alone, depending on the angiographic and clinical characteristics of the culprit lesion. The total number of newly implanted coronary stents during the index procedure was recorded. Balloon-only PCI without new stent implantation was performed when clinically appropriate, including selected cases involving culprit stent thrombosis or a small-caliber coronary vessel in which additional stent implantation was not considered appropriate.

Symptom onset-to-presentation time was defined as the interval between symptom onset and hospital arrival. Door-to-wire time was defined as the interval from hospital arrival to guidewire passage across the culprit lesion during primary PCI. Total ischemic time was calculated as the sum of symptom onset-to-presentation time and door-to-wire time.

Manual thrombus aspiration was selectively performed in patients with a high thrombus burden when considered necessary by the operating interventional cardiologist. Glycoprotein IIb/IIIa inhibitor therapy with tirofiban was administered at weight-adjusted doses in selected patients with distal coronary embolization, no-reflow, or a high thrombus burden and, when indicated, was continued as a maintenance infusion for 12–18 h. Procedural strategy, including vascular access, stent selection, and post-dilatation, was determined at the discretion of the operating interventional cardiologist.

Final antegrade flow in the infarct-related artery was assessed visually from the final post-PCI angiographic acquisition using the Thrombolysis in Myocardial Infarction (TIMI) flow-grade classification. Angiographic no-reflow was defined as final post-PCI TIMI flow grade < 3 (grades 0–2) despite restoration of epicardial patency and in the absence of an evident mechanical cause, such as flow-limiting residual stenosis, coronary dissection, or epicardial spasm. This variable was examined only as an exploratory procedural finding and was not a prespecified study objective.

### 2.8. Definition of Contrast-Associated Acute Kidney Injury

The primary endpoint of the study was the development of contrast-associated acute kidney injury (CA-AKI).

CA-AKI was defined using serum creatinine criteria consistent with the European Society of Urogenital Radiology (ESUR) recommendations: an increase in serum creatinine of ≥0.3 mg/dL or ≥1.5-fold from the baseline value within 48–72 h after contrast exposure [1]. Urine output data were unavailable for retrospective assessment; therefore, the urine output criterion was not applied, and CA-AKI ascertainment was based exclusively on serum creatinine criteria. Patients meeting either serum creatinine criterion were classified as having CA-AKI, whereas those meeting neither criterion were classified as not having CA-AKI.

### 2.9. Study Outcomes

The primary outcome was the development of CA-AKI after primary PCI. The main analysis evaluated the independent association between the CALLY index measured at hospital admission and this outcome.

Secondary analyses evaluated the discriminatory ability of the CALLY index for CA-AKI, determined an exploratory CALLY cut-off value, and compared its discriminatory performance with that of CRP, the C-reactive protein-to-albumin ratio (CAR), the neutrophil-to-lymphocyte ratio (NLR), and the systemic immune-inflammation index (SII).

### 2.10. Statistical Analysis

Continuous variables were summarized as mean ± standard deviation (SD) when approximately normally distributed and as median [interquartile range (IQR)] when skewed. Categorical variables were presented as frequencies and percentages. Comparisons between patients with and without CA-AKI were performed using Welch’s independent-samples *t*-test for approximately normally distributed continuous variables and the Mann–Whitney U test for non-normally distributed continuous variables. Categorical variables were compared using Pearson’s chi-square test or Fisher’s exact test, as appropriate.

Univariable logistic regression analyses were performed to evaluate variables associated with the development of CA-AKI. A multivariable logistic regression model was constructed to evaluate the independent association between the CALLY index and CA-AKI. Covariates were selected a priori based on clinical relevance and previously reported associations with CA-AKI, while limiting model complexity to reduce the risk of overfitting. The primary model included age, sex, hypertension, diabetes mellitus, baseline serum creatinine, hemoglobin, left ventricular ejection fraction (LVEF), Killip class ≥ 2, contrast volume, total ischemic time, the number of diseased major epicardial coronary vessels, and the CALLY index.

Age was expressed per 10-year increase, hemoglobin per 1-g/dL decrease, LVEF per 5-percentage-point decrease, contrast volume per 50-mL increase, and total ischemic time per 60-min increase. The number of diseased vessels was entered as an ordinal variable ranging from 1 to 3 and was expressed per one-vessel increase. Because the CALLY index showed a right-skewed distribution, it was natural-log transformed before inclusion in the regression model. To improve clinical interpretability, the regression coefficient for log-transformed CALLY was re-expressed as the odds ratio (OR) associated with each 50% decrease in the CALLY index.

The assumption of linearity in the logit was assessed for continuous covariates using a restricted cubic spline (natural cubic spline) basis with four degrees of freedom. Evidence of nonlinearity was assessed by a likelihood-ratio comparison between the spline model and the corresponding linear specification. Variables displaying significant nonlinearity were retained as spline terms in the primary multivariable model. Multicollinearity among the model covariates was assessed using variance inflation factors before spline expansion.

Baseline eGFR was excluded from the multivariable model because baseline serum creatinine was already included and the two variables are mathematically related measures of renal function. Similarly, CRP, serum albumin, lymphocyte count, CAR, NLR, and SII were not included in the primary multivariable model with CALLY because several of these markers overlap with components of the CALLY index, and the main objective was to evaluate the independent association between CALLY and CA-AKI. No-reflow was not included in the primary model because it occurred during the index procedure after baseline CALLY assessment and was considered a potential intermediate procedural event rather than a baseline confounder. Peak post-contrast serum creatinine was not included because it formed part of the CA-AKI endpoint definition.

Receiver operating characteristic (ROC) curve analysis was performed to assess the ability of the CALLY index, CAR, CRP, NLR, and SII to discriminate between patients with and without CA-AKI. Because lower CALLY values indicate greater risk, the direction of the CALLY ROC curve was reversed accordingly. In contrast, higher values of CAR, CRP, NLR, and SII were considered indicative of greater risk. Discriminatory performance was quantified using the area under the ROC curve (AUC) with 95% confidence intervals (CIs). The 95% CIs for the AUCs were estimated using 5000 stratified bootstrap resamples.

The AUC of the CALLY index was compared separately with those of CAR, CRP, NLR, and SII using DeLong’s nonparametric test for correlated ROC curves [18]. To account for the four pairwise comparisons, *p*-values were adjusted using the Holm method; both unadjusted and Holm-adjusted *p*-values were reported, and statistical significance for these comparisons was determined using the Holm-adjusted *p*-values.

Optimal cut-off values for each marker were determined using the maximum Youden index. Sensitivity, specificity, positive predictive value (PPV), and negative predictive value (NPV) were calculated at the corresponding thresholds. Because the optimal thresholds were derived and evaluated within the same study cohort, all cut-off analyses were considered exploratory and were not interpreted as externally validated clinical thresholds.

No a priori sample size calculation was performed; all eligible patients during the study period were included. The final cohort comprised 903 patients, including 139 CA-AKI events. The multivariable model included 15 predictor parameters, excluding the intercept, with four degrees of freedom for baseline serum creatinine modeled using a restricted cubic spline. This yielded approximately 9.3 events per predictor parameter.

**Figure 1:**
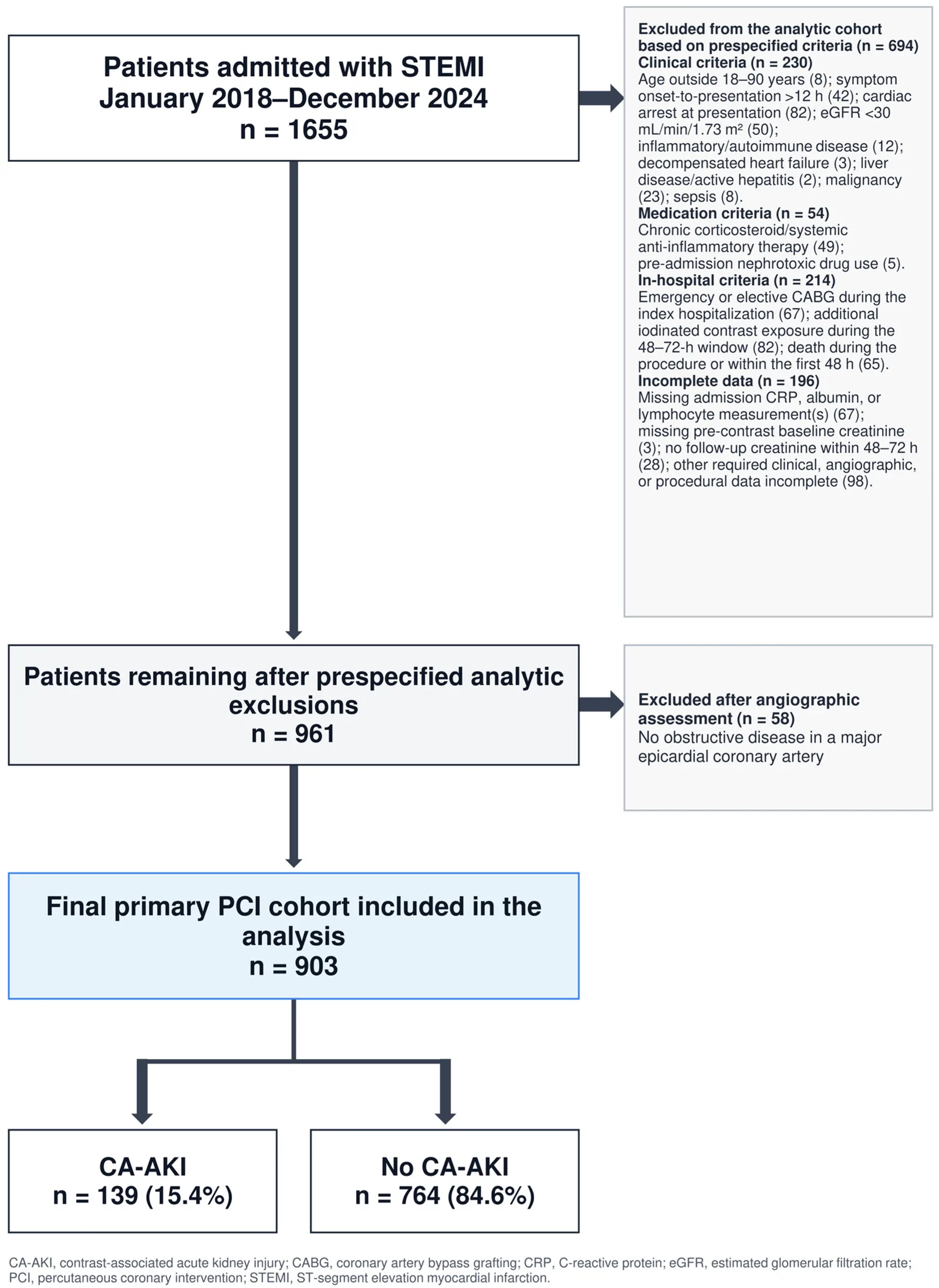
Study flow diagram.

Patients with incomplete data required for eligibility, calculation of the study indices, or ascertainment of the primary endpoint were excluded during cohort construction, as detailed in Figure 1. The final analytic cohort had no missing observations for the primary endpoint or variables included in the reported analyses; therefore, no imputation was performed. All statistical tests were two-sided. A *p*-value < 0.05 was considered statistically significant; for the four pairwise ROC comparisons, significance was determined using Holm-adjusted *p*-values. Statistical analyses were performed using Python version 3.13 with SciPy version 1.18.0, statsmodels version 0.14.6, scikit-learn version 1.9.0, and Patsy version 1.0.2.

## 3. Results

### 3.1. Study Population and Baseline Characteristics

During the study period, 1655 patients admitted with STEMI were screened. Of these, 694 were excluded based on the prespecified exclusion criteria, leaving 961 patients. A further 58 patients were excluded after angiographic assessment because no obstructive disease was identified in a major epicardial coronary artery. The final analysis included 903 patients, of whom 139 (15.4%) developed CA-AKI and 764 (84.6%) did not. Patient selection is summarized in Figure 1.

Patients who developed CA-AKI were older than those without CA-AKI (67.6 ± 11.6 vs. 57.4 ± 11.1 years, *p* < 0.001) and were more frequently female (37.4% vs. 17.4%, *p* < 0.001). Hypertension (61.2% vs. 38.7%, *p* < 0.001), diabetes mellitus (44.6% vs. 25.1%, *p* < 0.001), and Killip class ≥ 2 at presentation (44.6% vs. 33.0%, *p* = 0.008) were also more frequent in the CA-AKI group. Pre-admission ACEI/ARB use was also more frequent in the CA-AKI group (43.2% vs. 29.8%, *p* = 0.002).

Patients with CA-AKI had higher baseline serum creatinine levels (1.06 [0.85–1.29] vs. 0.86 [0.76–0.99] mg/dL, *p* < 0.001) and lower baseline eGFR (69.8 ± 26.0 vs. 93.2 ± 17.7 mL/min/1.73 m^2^, *p* < 0.001). Serum albumin (36.7 ± 4.4 vs. 39.2 ± 3.6 g/L, *p* < 0.001), hemoglobin (12.8 ± 2.1 vs. 14.0 ± 1.7 g/dL, *p* < 0.001), and LVEF (43.1 ± 11.5% vs. 49.6 ± 10.1%, *p* < 0.001) were lower in patients who developed CA-AKI.

The CALLY index was markedly lower in patients with CA-AKI than in those without CA-AKI (0.635 [0.193–1.529] vs. 1.536 [0.891–2.356], *p* < 0.001). In contrast, CAR (0.193 [0.095–0.904] vs. 0.092 [0.081–0.186], *p* < 0.001), CRP (7.25 [3.23–31.70] vs. 3.34 [3.22–7.06] mg/L, *p* < 0.001), NLR (6.54 [3.19–10.09] vs. 5.05 [3.15–7.42], *p* = 0.001), and SII (1514.3 [762.0–2419.0] vs. 1138.5 [722.1–1727.0], *p* = 0.002) were higher in the CA-AKI group.

Symptom onset-to-presentation time (160 [108–222] vs. 134 [101–180] min, *p* = 0.014) and total ischemic time (205 [146–266] vs. 180 [141–236] min, *p* = 0.029) were longer among patients who developed CA-AKI, whereas door-to-wire time did not differ significantly between groups (*p* = 0.443). Total contrast volume was also similar between groups (190 [140–250] vs. 180 [130–230] mL, *p* = 0.213).

The number of diseased major epicardial coronary vessels was higher in patients with CA-AKI (2 [1–2] vs. 1 [1–2], *p* < 0.001). The SYNTAX score showed a nonsignificant trend toward higher values in the CA-AKI group (17.0 [10.8–25.2] vs. 15.0 [10.0–21.5], *p* = 0.055). In an exploratory unadjusted comparison, angiographic no-reflow occurred more frequently in patients who developed CA-AKI than in those who did not (20.9% vs. 11.9%, *p* = 0.004). No-reflow was not included in the primary multivariable model because it occurred during the index procedure after baseline CALLY assessment and was considered a potential intermediate procedural event rather than a baseline confounder.

The distribution of the infarct-related artery did not differ significantly between groups (*p* = 0.128), and the number of newly implanted stents was similar (*p* = 0.832). Baseline demographic, clinical, laboratory, angiographic, and procedural characteristics are summarized in Table 1.

### 3.2. Association Between the CALLY Index and CA-AKI

Assessment of the linearity assumption demonstrated a nonlinear association between baseline serum creatinine and the log-odds of CA-AKI (*p* for nonlinearity = 0.009); therefore, baseline creatinine was modeled using a restricted cubic spline with four degrees of freedom. No evidence of significant nonlinearity was observed for age, hemoglobin, LVEF, contrast volume, total ischemic time, or log-transformed CALLY (all *p* for nonlinearity ≥ 0.135). There was no evidence of problematic multicollinearity among the model covariates before spline expansion (all VIFs < 2).

In univariable logistic regression analysis, each 50% decrease in the CALLY index was associated with an 89.1% increase in the odds of CA-AKI (OR 1.891, 95% CI 1.662–2.151; *p* < 0.001).

After adjustment for age, sex, hypertension, diabetes mellitus, baseline serum creatinine, hemoglobin, LVEF, Killip class ≥ 2, contrast volume, total ischemic time, and diseased-vessel count, a lower CALLY index remained independently associated with CA-AKI. Each 50% decrease in CALLY was associated with a 58.7% increase in the adjusted odds of CA-AKI (adjusted OR 1.587, 95% CI 1.367–1.843; *p* < 0.001). Baseline serum creatinine, modeled as a restricted cubic spline, was also significantly associated with CA-AKI overall (*p* < 0.001).

Other variables independently associated with CA-AKI were older age (adjusted OR per 10-year increase 1.433, 95% CI 1.150–1.785; *p* = 0.001), lower LVEF (adjusted OR per 5-percentage-point decrease 1.207, 95% CI 1.087–1.341; *p* < 0.001), longer total ischemic time (adjusted OR per 60-min increase 1.135, 95% CI 1.012–1.272; *p* = 0.030), and greater angiographic disease extent (adjusted OR per one-vessel increase 1.457, 95% CI 1.078–1.971; *p* = 0.015). Female sex, hypertension, diabetes mellitus, hemoglobin, Killip class ≥ 2, and contrast volume were not independently associated with CA-AKI after multivariable adjustment. Complete univariable and multivariable results are presented in Table 2.

### 3.3. Discriminatory Performance of CALLY and Other Inflammatory Markers

In ROC curve analysis, the CALLY index showed the highest numerical AUC for CA-AKI, with an AUC of 0.734 (95% CI 0.683–0.782). The AUCs were 0.707 (95% CI 0.654–0.759) for CAR, 0.674 (95% CI 0.615–0.730) for CRP, 0.587 (95% CI 0.531–0.644) for NLR, and 0.582 (95% CI 0.526–0.639) for SII.

In pairwise DeLong comparisons, the discriminatory performance of CALLY was not significantly different from that of CAR (ΔAUC = 0.026, unadjusted *p* = 0.163; Holm-adjusted *p* = 0.163). CALLY demonstrated significantly greater discrimination than CRP (ΔAUC = 0.059, unadjusted *p* = 0.004; Holm-adjusted *p* = 0.008), NLR (ΔAUC = 0.147, unadjusted *p* < 0.001; Holm-adjusted *p* < 0.001), and SII (ΔAUC = 0.151, unadjusted *p* < 0.001; Holm-adjusted *p* < 0.001).

**Figure 2:**
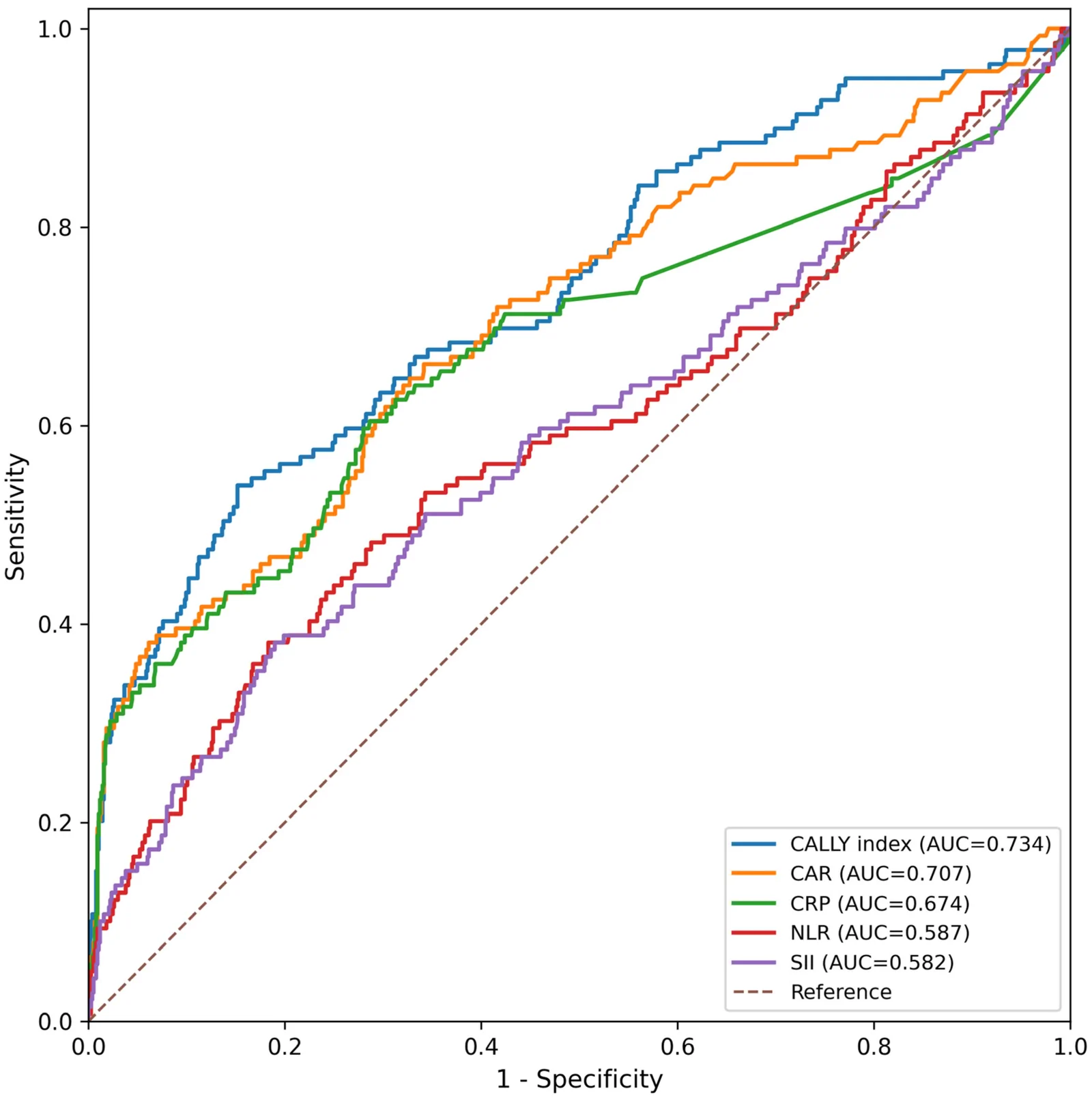
Receiver operating characteristic curves of the CALLY index, CAR, CRP, NLR, and SII for discrimination of contrast-associated acute kidney injury. CALLY showed an AUC of 0.734, compared with 0.707 for CAR, 0.674 for CRP, 0.587 for NLR, and 0.582 for SII. The dashed diagonal line represents the reference AUC of 0.50. CALLY, C-reactive protein–albumin–lymphocyte index; CAR, C-reactive protein-to-albumin ratio; CRP, C-reactive protein; NLR, neutrophil-to-lymphocyte ratio; SII, systemic immune-inflammation index; AUC, area under the curve.

The exploratory optimal CALLY threshold identified by the Youden index was ≤0.697, yielding a sensitivity of 54.0%, specificity of 84.8%, PPV of 39.3%, and NPV of 91.0% for CA-AKI. Detailed ROC characteristics for all markers are presented in Table 3, pairwise comparisons are provided in Supplementary Table S1, and the ROC curves are shown in Figure 2.

## 4. Discussion

In this cohort, a lower C-reactive protein–albumin–lymphocyte (CALLY) index at admission was independently associated with contrast-associated acute kidney injury (CA-AKI) in patients with ST-segment elevation myocardial infarction (STEMI) treated with primary percutaneous coronary intervention (PCI). The association persisted after adjustment for age, baseline renal function, left ventricular ejection fraction (LVEF), Killip class, contrast volume, total ischemic time, and the extent of coronary artery disease. In practical terms, each 50% decrease in CALLY was associated with a 58.7% increase in the adjusted odds of CA-AKI. CALLY also showed moderate discriminatory ability (AUC 0.734) and the highest numerical AUC among the inflammatory indices examined. It discriminated CA-AKI significantly better than CRP, NLR, and SII, but not better than the CRP-to-albumin ratio (CAR). The results support further evaluation of CALLY as a potential adjunct to conventional risk assessment rather than as a stand-alone prediction tool.

CA-AKI occurred in 15.4% of our cohort. This rate is somewhat higher than the approximately 12% incidence reported in a meta-analysis of more than 22,000 patients with STEMI undergoing primary PCI [2]. Such variation is not unexpected. Differences in patient characteristics, renal endpoint definitions, ischemic and hemodynamic burden, and local procedural practice can each influence the observed incidence of CA-AKI.

The current results add to the growing cardiovascular literature on CALLY. Recently, lower CALLY values have been associated with in-hospital mortality after primary PCI for STEMI [10]. Regarding renal outcomes, Koç et al. retrospectively screened 1308 patients who underwent elective coronary angiography for stable angina pectoris and included 945 patients in the final analysis; CA-AKI developed in 150 patients (15.9%), and a lower CALLY index remained independently associated with CA-AKI in the multivariable model, although its discriminatory ability was modest (AUC 0.618) [11]. Gul et al. retrospectively evaluated 199 consecutive patients with NSTEMI undergoing PCI, among whom 32 (16.1%) developed contrast-induced nephropathy. In that study, lower CALLY values remained independently associated with renal injury after multivariable adjustment and showed moderate discrimination (AUC 0.697) [12].

Neither study specifically evaluated CA-AKI in patients with STEMI undergoing primary PCI. STEMI treated with primary PCI represents a particularly acute clinical setting in which extensive ischemic injury, marked inflammatory activation, hemodynamic stress, and urgent contrast exposure may occur simultaneously. In addition, differences in renal endpoint definitions limit direct comparison of discriminatory performance among studies. To our knowledge, this is the first study to specifically evaluate the association between CALLY and CA-AKI in patients with STEMI undergoing primary PCI.

CA-AKI is multifactorial, involving renal vasoconstriction, medullary hypoxia, oxidative stress, endothelial and tubular injury, and inflammatory activation [1,3,19]. In STEMI, several of these processes may already be active before contrast is administered. The three components of CALLY may therefore capture complementary aspects of renal vulnerability. CRP reflects systemic inflammatory activity; albumin is influenced by inflammation, nutritional reserve, hepatic synthesis, and intravascular volume status; and lymphopenia may accompany acute stress and altered immune homeostasis. A composite measure incorporating all three may reflect the patient’s inflammatory, immune, and nutritional status more comprehensively than any single component.

Previous work in acute coronary syndromes supports this broader concept. In STEMI treated with primary PCI, inflammation-dominant indices, including NLR and SII, have been associated with post-contrast renal injury [4,7]. In contrast, markers that incorporate nutritional or immune information, including CAR, the prognostic nutritional index, and the lymphocyte-to-CRP ratio, have also shown significant associations [5,6,8]. More recent studies have extended this approach to other composite markers. Yildiz et al. reported strong discrimination with SII and the systemic inflammation response index in selected elderly patients with STEMI [20]; Guler et al. found the albumin-bilirubin score to be independently associated with contrast-induced acute kidney injury in 2011 patients with STEMI undergoing primary PCI [21], and Konte et al. described an association between the neutrophil percentage-to-albumin ratio and renal injury after PCI in acute coronary syndrome [22]. In a large 2026 cohort of 2325 patients with STEMI undergoing primary PCI, Çiçek et al. also found that the pan-immune-inflammation value (PIV), which integrates neutrophil, platelet, monocyte, and lymphocyte counts, was independently associated with study-defined CA-AKI and showed an AUC of 0.753 [23]. Because that study used a different CA-AKI definition, direct comparison of AUC values is limited.

The comparison with CAR is particularly informative. CALLY had a numerically higher AUC than CAR (0.734 vs. 0.707), but the difference was not statistically significant (DeLong *p* = 0.163). Therefore, the present findings do not demonstrate superior discriminatory performance of CALLY over CAR. In contrast, CALLY showed significantly greater discrimination than CRP, NLR, and SII. Overall, CALLY demonstrated moderate discriminatory ability for CA-AKI, although a significant advantage over CAR was not established in this cohort.

Several conventional risk factors also remained independently associated with CA-AKI. Older age, lower LVEF, longer total ischemic time, greater angiographic disease extent, and baseline serum creatinine remained independently associated with CA-AKI in the adjusted model. This pattern is broadly consistent with previous STEMI data. In the meta-analysis by Ye et al., older age, diabetes, heart failure-related variables, multivessel coronary disease, and higher contrast exposure were among the factors associated with CA-AKI [2]. Contrast volume was not independently associated with CA-AKI in our adjusted model. This finding should not be interpreted as evidence contrary to the potential renal effects of contrast exposure. Still, it may reflect adjustment for correlated clinical factors and the multifactorial nature of CA-AKI. The independent association with diseased-vessel count is likewise consistent with the reported relationship between multivessel coronary disease and CA-AKI [2].

Angiographic no-reflow was more frequent among patients who subsequently developed CA-AKI than among those who did not (20.9% vs. 11.9%, *p* = 0.004). Previous studies have associated impaired baseline renal function with the slow-flow/no-reflow phenomenon in STEMI [24] and have evaluated CALLY as a predictor of no-reflow in patients with STEMI undergoing PCI [25]. However, no-reflow was an exploratory procedural outcome in the present study and was not evaluated in a dedicated adjusted model; therefore, this observation should not be interpreted as evidence of an independent association between no-reflow and CA-AKI or of causality.

The main practical appeal of CALLY is its simplicity. It can be calculated from commonly available laboratory parameters, without the need for a dedicated biomarker assay. Its performance in this study, however, was moderate rather than excellent: the AUC was 0.734, and the exploratory cut-off of ≤0.697 favored specificity over sensitivity. CALLY should therefore not replace clinical judgment or established multivariable risk assessment. A recent systematic review of CA-AKI prediction models in STEMI after PCI reported AUC/C-index values ranging from 0.719 to 0.877. However, most models were considered at high risk of bias, and their clinical utility remained uncertain [26]. Those multivariable models are not directly comparable with the stand-alone AUC of a single composite biomarker such as CALLY. If prospectively validated, a low admission CALLY index could complement established clinical risk assessment by identifying patients who may warrant closer renal monitoring after primary PCI. Its potential role in informing individualized hydration and contrast-minimization strategies merits further investigation.

### Study Limitations

Several limitations should be considered when interpreting the results. First, the retrospective, single-center design leaves the study vulnerable to residual confounding, selection bias, and limitations inherent to routinely collected clinical data. Moreover, exclusion of 196 patients with incomplete required data may have introduced additional selection bias if missingness was not random. Patient-level data on other concomitant medications were unavailable for analysis; therefore, residual confounding related to these treatments cannot be excluded. Second, patients with an eGFR < 30 mL/min/1.73 m^2^ and several conditions that could substantially alter inflammatory or nutritional biomarkers were excluded, which limits generalizability to patients with advanced chronic kidney disease and other excluded populations. In addition, patients who died during the index procedure or within the first 48 h were excluded because follow-up creatinine measurements required for CA-AKI ascertainment were unavailable; this may have introduced survivor bias and excluded some of the highest-risk patients. Third, the lower analytical limit of CRP at our institution was 3 mg/L, and values under this threshold were assigned 3 mg/L. This may have reduced discrimination among patients with low inflammatory activity and may have affected both CALLY and CAR calculations. Fourth, because CALLY was calculated from admission laboratory measurements obtained during the acute STEMI presentation, we cannot separate chronic inflammatory or nutritional vulnerability from the acute-phase response to myocardial infarction. Fifth, CA-AKI was defined as a change in serum creatinine within 48–72 h. Because urine output criteria could not be assessed, AKI meeting only the oliguria criterion may have been missed. Serum creatinine is an established but delayed and imperfect marker of acute renal injury, and the observational design cannot determine the extent to which renal dysfunction was directly attributable to contrast exposure. Sixth, the optimal CALLY cut-off was derived and tested in the same cohort and should therefore be viewed as exploratory until externally validated. Although the SYNTAX score and angiographic no-reflow were assessed retrospectively and independently by two cardiologists blinded to the patients’ CALLY values and CA-AKI status, with disagreements resolved by consensus, formal interobserver agreement statistics were not available; therefore, these angiographic assessments remain susceptible to observer-related variability, and the no-reflow findings should be interpreted as exploratory. The number of CA-AKI events relative to the complexity of the multivariable model was modest. Although covariates were selected based on clinical relevance, overfitting and instability of the adjusted estimates cannot be excluded, and confirmation in independent cohorts is required. Finally, serial CALLY measurements were not available. Long-term outcomes, including mortality and the need for renal replacement therapy, were not evaluated. Therefore, the association of admission CALLY with long-term renal outcomes and survival could not be assessed. Prospective multicenter studies should replicate these findings in larger patient cohorts and clarify the clinical utility of CALLY for CA-AKI risk assessment.

## 5. Conclusions

In conclusion, a lower admission CALLY index was independently associated with CA-AKI in patients with STEMI undergoing primary PCI after multivariable adjustment. CALLY showed significantly greater discrimination for CA-AKI than CRP, NLR, and SII, whereas its discriminatory performance was not significantly different from that of CAR. As a readily calculable composite marker derived from routinely available laboratory parameters, CALLY warrants further evaluation as a potential adjunct to CA-AKI risk assessment. Prospective multicenter studies are needed to validate these findings and clarify the clinical role of CALLY in this setting.

## Figures and Tables

**Table 1:** Baseline Clinical, Laboratory, Angiographic, and Procedural Characteristics According to CA-AKI Status.

Variable	Overall (*n* = 903)	No CA-AKI (*n* = 764)	CA-AKI (*n* = 139)	*p*-Value
**Demographic and clinical characteristics**				
Age, years	59.0 ± 11.8	57.4 ± 11.1	67.6 ± 11.6	<0.001
Female sex, *n* (%)	185 (20.5)	133 (17.4)	52 (37.4)	<0.001
Hypertension, *n* (%)	381 (42.2)	296 (38.7)	85 (61.2)	<0.001
Diabetes mellitus, *n* (%)	254 (28.1)	192 (25.1)	62 (44.6)	<0.001
Smoking, *n* (%)	572 (63.3)	517 (67.7)	55 (39.6)	<0.001
Dyslipidemia, *n* (%)	100 (11.1)	88 (11.5)	12 (8.6)	0.319
Previous cerebrovascular disease, *n* (%)	8 (0.9)	4 (0.5)	4 (2.9)	0.023
Previous coronary artery disease, *n* (%)	116 (12.8)	85 (11.1)	31 (22.3)	<0.001
Killip class ≥ 2, *n* (%)	314 (34.8)	252 (33.0)	62 (44.6)	0.008
Pre-admission ACEI/ARB use, *n* (%)	288 (31.9)	228 (29.8)	60 (43.2)	0.002
**Laboratory and echocardiographic characteristics**				
Baseline creatinine, mg/dL	0.89 [0.77–1.03]	0.86 [0.76–0.99]	1.06 [0.85–1.29]	<0.001
Baseline eGFR, mL/min/1.73 m^2^	89.6 ± 21.0	93.2 ± 17.7	69.8 ± 26.0	<0.001
Albumin, g/L	38.8 ± 3.8	39.2 ± 3.6	36.7 ± 4.4	<0.001
Lymphocyte count, ×10^9^/L	1.74 [1.23–2.38]	1.79 [1.30–2.40]	1.47 [0.94–2.23]	<0.001
CRP, mg/L	3.69 [3.22–7.95]	3.34 [3.22–7.06]	7.25 [3.23–31.70]	<0.001
CAR	0.098 [0.081–0.207]	0.092 [0.081–0.186]	0.193 [0.095–0.904]	<0.001
NLR	5.14 [3.15–7.95]	5.05 [3.15–7.42]	6.54 [3.19–10.09]	0.001
SII	1180.9 [728.9–1833.3]	1138.5 [722.1–1727.0]	1514.3 [762.0–2419.0]	0.002
CALLY index	1.412 [0.780–2.244]	1.536 [0.891–2.356]	0.635 [0.193–1.529]	<0.001
White blood cell count, ×10^9^/L	11.42 [9.40–14.27]	11.42 [9.43–14.23]	11.40 [9.12–14.70]	0.919
Neutrophil count, ×10^9^/L	8.62 [6.60–11.33]	8.57 [6.63–11.15]	8.94 [6.44–12.38]	0.216
Hemoglobin, g/dL	13.8 ± 1.8	14.0 ± 1.7	12.8 ± 2.1	<0.001
Platelet count, ×10^9^/L	232.0 [198.0–270.0]	232.5 [195.8–268.0]	228.0 [206.0–273.5]	0.677
LVEF, %	48.6 ± 10.6	49.6 ± 10.1	43.1 ± 11.5	<0.001
**Angiographic and procedural characteristics**				
SYNTAX score	15.0 [10.0–22.5]	15.0 [10.0–21.5]	17.0 [10.8–25.2]	0.055
Number of diseased vessels	1 [1–2]	1 [1–2]	2 [1–2]	<0.001
Infarct-related artery, *n* (%)				0.128
LAD	346 (38.3)	287 (37.6)	59 (42.4)	
LCx	161 (17.8)	139 (18.2)	22 (15.8)	
RCA	363 (40.2)	314 (41.1)	49 (35.3)	
Other (side branches)	33 (3.7)	24 (3.1)	9 (6.5)	
Contrast volume, mL	180 [130–240]	180 [130–230]	190 [140–250]	0.213
Number of newly implanted stents	1 [1–1]	1 [1–1]	1 [1–2]	0.832
P2Y12 inhibitor, *n* (%)				0.024
Clopidogrel	346 (38.3)	283 (37.0)	63 (45.3)	
Ticagrelor	467 (51.7)	397 (52.0)	70 (50.4)	
Prasugrel	90 (10.0)	84 (11.0)	6 (4.3)	
Post-dilatation, *n* (%)	429 (47.5)	372 (48.7)	57 (41.0)	0.095
Angiographic no-reflow, *n* (%)	120 (13.3)	91 (11.9)	29 (20.9)	0.004
Symptom onset-to-presentation time, min	136 [102–180]	134 [101–180]	160 [108–222]	0.014
Door-to-wire time, min	39 [29–56]	39 [29–55]	42 [28–57]	0.443
Total ischemic time, min	185 [142–246]	180 [141–236]	205 [146–266]	0.029

Data are presented as mean ± standard deviation, median [interquartile range], or *n* (%). Welch’s *t*-test, Mann–Whitney U test, Pearson chi-square test, or Fisher’s exact test was used as appropriate. ACEI, angiotensin-converting enzyme inhibitor; ARB, angiotensin II receptor blocker; CA-AKI, contrast-associated acute kidney injury; eGFR, estimated glomerular filtration rate; CRP, C-reactive protein; CAR, C-reactive protein-to-albumin ratio; NLR, neutrophil-to-lymphocyte ratio; SII, systemic immune-inflammation index; CALLY, C-reactive protein–albumin–lymphocyte index; LVEF, left ventricular ejection fraction; LAD, left anterior descending artery; LCx, left circumflex artery; RCA, right coronary artery. P2Y12, platelet adenosine diphosphate P2Y12 receptor.

**Table 2:** Univariable and Multivariable Logistic Regression Analyses for CA-AKI.

Variable	Unadjusted OR (95% CI)	*p*-Value	Adjusted OR (95% CI)	*p*-Value
Age, per 10-year increase	2.185 (1.837–2.600)	<0.001	1.433 (1.150–1.785)	0.001
Female sex	2.836 (1.918–4.193)	<0.001	1.468 (0.819–2.629)	0.197
Hypertension	2.489 (1.718–3.606)	<0.001	1.096 (0.668–1.798)	0.717
Diabetes mellitus	2.399 (1.653–3.481)	<0.001	1.354 (0.831–2.208)	0.224
Baseline serum creatinine	Restricted cubic spline (4 df)	<0.001	Restricted cubic spline (4 df)	<0.001
Hemoglobin, per 1-g/dL decrease	1.456 (1.317–1.610)	<0.001	1.057 (0.923–1.210)	0.423
LVEF, per 5-percentage-point decrease	1.333 (1.222–1.453)	<0.001	1.207 (1.087–1.341)	<0.001
Killip class ≥ 2	1.636 (1.134–2.361)	0.009	1.534 (0.912–2.580)	0.107
Contrast volume, per 50-mL increase	1.109 (0.996–1.236)	0.060	1.030 (0.897–1.182)	0.678
Total ischemic time, per 60-min increase	1.096 (1.006–1.195)	0.035	1.135 (1.012–1.272)	0.030
Diseased-vessel count, per 1-vessel increase	1.794 (1.405–2.290)	<0.001	1.457 (1.078–1.971)	0.015
CALLY index, per 50% decrease	1.891 (1.662–2.151)	<0.001	1.587 (1.367–1.843)	<0.001

Baseline serum creatinine demonstrated a nonlinear association with CA-AKI and was therefore modeled using a restricted cubic spline (natural cubic spline basis) with four degrees of freedom in both univariable and multivariable analyses; a single OR is not reported for this variable. In the adjusted model, *p* for nonlinearity was 0.009 and the overall association was *p* < 0.001. Diseased-vessel count was entered as an ordinal variable from 1 to 3 and is reported per one-vessel increase. CALLY was natural-log transformed and is reported as the OR associated with each 50% decrease. The multivariable model included all variables shown in the table. OR, odds ratio; CI, confidence interval; LVEF, left ventricular ejection fraction; CALLY, C-reactive protein–albumin–lymphocyte index.

**Table 3:** ROC Analysis of CALLY, CAR, CRP, NLR, and SII for Discrimination of CA-AKI.

Marker	AUC	95% CI	Optimal Cut-Off	Sensitivity	Specificity	PPV	NPV	DeLong *p* vs. CALLY	Holm-Adjusted *p*
CALLY index	0.734	0.683–0.782	≤0.697	54.0%	84.8%	39.3%	91.0%	Reference	Reference
CAR	0.707	0.654–0.759	≥0.137	64.7%	67.3%	26.5%	91.3%	0.163	0.163
CRP	0.674	0.615–0.730	≥6.30	60.4%	71.3%	27.7%	90.8%	0.004	0.008
NLR	0.587	0.531–0.644	≥8.417	38.1%	81.7%	27.5%	87.9%	<0.001	<0.001
SII	0.582	0.526–0.639	≥1936	38.1%	81.0%	26.8%	87.8%	<0.001	<0.001

Lower CALLY values and higher CAR, CRP, NLR, and SII values were considered indicative of higher CA-AKI risk. Optimal cut-offs were determined using the maximum Youden index. AUC 95% CIs were estimated using 5000 stratified bootstrap resamples. Pairwise comparisons with CALLY were performed using DeLong’s test for correlated ROC curves; Holm adjustment was applied for four comparisons. Cut-offs are exploratory because they were derived and evaluated in the same cohort. AUC, area under the curve; PPV, positive predictive value; NPV, negative predictive value.

## Data Availability

De-identified data supporting the findings of this study may be made available by the corresponding author upon reasonable request, subject to institutional approval and applicable ethical and privacy restrictions. The data are not publicly available because the source dataset contains potentially identifiable patient information.

## Supplementary Materials

The following supporting information can be downloaded at: https://www.mdpi.com/article/10.3390/medicina62091811/s1, Supplementary Table S1: Pairwise Comparison of ROC AUCs.

## Abbreviations

The following abbreviations are used in this manuscript:

ACEI	angiotensin-converting enzyme inhibitor
ARB	angiotensin II receptor blocker
AUC	area under the receiver operating characteristic curve
CA-AKI	contrast-associated acute kidney injury
CABG	coronary artery bypass grafting
CALLY	C-reactive protein–albumin–lymphocyte index
CAR	C-reactive protein-to-albumin ratio
CI	confidence interval
CKD-EPI	Chronic Kidney Disease Epidemiology Collaboration
CRP	C-reactive protein
eGFR	estimated glomerular filtration rate
ESUR	European Society of Urogenital Radiology
IQR	interquartile range
IRA	infarct-related artery
LAD	left anterior descending artery
LCx	left circumflex artery
LVEF	left ventricular ejection fraction
NLR	neutrophil-to-lymphocyte ratio
NPV	negative predictive value
NSTEMI	non-ST-segment elevation myocardial infarction
OR	odds ratio
P2Y12	platelet adenosine diphosphate P2Y12 receptor
PCI	percutaneous coronary intervention
PIV	pan-immune-inflammation value
PPV	positive predictive value
RCA	right coronary artery
ROC	receiver operating characteristic
SD	standard deviation
SII	systemic immune-inflammation index
STEMI	ST-segment elevation myocardial infarction
STROBE	Strengthening the Reporting of Observational Studies in Epidemiology
SYNTAX	Synergy between Percutaneous Coronary Intervention with Taxus and Cardiac Surgery
TIMI	Thrombolysis in Myocardial Infarction
VIF	variance inflation factor
