# Supplementary material for: Lower Admission CALLY Index Is Associated with Contrast-Associated Acute Kidney Injury in Patients with STEMI Undergoing Primary PCI
Source: Medicina (Kaunas). 2026 Sep 20;62(9):1811. doi: 10.3390/medicina62091811 (PMC13609819; doi:10.3390/medicina62091811)
Supplement: Supplementary file 1 [file medicina-62-01811-s001.zip › medicina-4550434-supplementary.pdf]

Supplementary Table S1. Pairwise Comparison of ROC AUCs

Comparison	ΔAUC	z statistic	Unadjusted p value	Holm-adjusted p value
CALLY vs. CAR	0.026	1.395	0.163	0.163
CALLY vs. CRP	0.059	2.862	0.004	0.008
CALLY vs. NLR	0.147	4.831	<0.001	<0.001
CALLY vs. SII	0.151	4.795	<0.001	<0.001

Pairwise comparisons of AUCs were performed using DeLong's test for correlated ROC curves. Holm adjustment was applied to account for four comparisons of CALLY with CAR, CRP, NLR, and SII. ΔAUC represents the difference between the AUC of CALLY and the comparator marker; positive values favor CALLY. AUC, area under the receiver operating characteristic curve; CALLY, C-reactive protein-albumin-lymphocyte index; CAR, C-reactive protein-to-albumin ratio; CRP, C-reactive protein; NLR, neutrophil-to-lymphocyte ratio; SII, systemic immune-inflammation index.
